# Supplementary material for: Peripheral anatomy of the dolphin ear and associated nervous structures: Insights from macroscopic dissection, DICE-µCT, histology, and confocal microscopy
Source: PLoS One. 2026 Aug 19;21(8):e0350484. doi: 10.1371/journal.pone.0350484 (PMC13489439; doi:10.1371/journal.pone.0350484)
Supplement: Supplementary file 3 [file pone.0350484.s004.docx]

Answer to reviewers

*Reviewer #1: I found the paper to be very interesting and a much needed description of the outer ear of odontocetes, which is an anatomical region that has received little attention. I particularly appreciate the discussion of the potential function of the outer ear region, which often is considered functionless.

Overall, the paper is well written and the conclusions are well supported by the observations presented. The figures are good, although I did find some of them a little difficult to interpret. In fact, the vast majority of my comments deal with the figures and associated captions themselves. I have made several comments and suggestions below, both general and specific, all of which I think will strengthen the paper.

A general comment about the figures is that a lot of the anatomy is difficult to interpret. Figures 4 & 5 were probably the most helpful, but I think that an illustrated or schematic figure that represents the 3-dimensional shapes would be very helpful. I also found the numbers in the figure to be a bit distracting and it was a little cumbersome to flick back and forth between the figure and the caption. One option would be to replace them with abbreviations, but that might be a matter of taste. I found the numbering to be especially distracting when they aren’t all listed numerically at the end of the captions, such as in Figure 3 in which some of the structures are numbered in the text of the caption. I would recommend listing all of the numbers at the end of the caption even if they are listed earlier in the caption text.*

*I also think that more specific citations to the figures would help the reader to follow the anatomical descriptions, especially in the ‘Mandibular fat bodies’ section beginning around page 11. When possible, please cite the figures more frequently.*

**Response**: Thank you for the suggestions, indeed it is a complex anatomical region that requires simplification of 3-dimensional shapes to guide the reader for orientation and identification of the anatomical structures of interest. We have added a simplified drawing of the anatomical components from the DICE-µCT study. We hope this helps for clarification of the topographical relationship between fat bodies and nerves. Also, the entire manuscript has been updated to include a single naming convention for each structure, to avoid the reader to hop back and forth for number identification in the figures. Additional citations to figures have been added throughout the text, and in the mandibular fat body section.

*Page 3, second sentence under ‘Materials and Methods’ - I think that “spotted dolphin foetus" should be plural here. (E.g., “two bottlenose dolphin…and Pantropical spotted dolphin foetuses.”)*

**Response**: That macroscopic dissection sample came from a single spotted dolphin foetus; sentence has been adjusted accordingly; and ‘single’ was removed from the last sentence of the paragraph to avoid repetition.

*Further in the same paragraph, “details” in “details can be found” should be lowercase.*

**Response**: Adjusted accordingly.

*First sentence of third paragraph under ‘Material and Methods’, “from the out ear canal” should be “from the outer ear canal”.*

**Response**: Adjusted accordingly.

*Page 5, first paragraph under ‘Region of the ear canal and middle ear’ - the anatomy described here is difficult to interpret even using the figures. This place in particular would benefit from a schematic or illustrated figure to orient the reader and accentuate the anatomy.*

**Response**: An illustrated simplified figure has been added to orient the reader, Figure 05.

*Page 6, Figure 1 caption - In case I missed it, IMFB and EMFB have not been defined by this point in the manuscript. These should be defined (i.e., spelled out) earlier in the manuscript.*

**Response**: Thank you for pointing this out. The spelled-out words and abbreviations have been added to the paragraph before Figure 1, with specific references to the structure identifications in the figure.

*Page 6, Figure 2 caption - Define/ spell out “TP-complex”. I am guessing this is the tympanoperiotic complex.*

**Response**: TP-complex is now spelled out, with abbreviation ‘TPC’, and also adjusted throughout the manuscript.

*Figure 3 - It could be on account of the resolution for the review, but the yellow asterisk was difficult to see. Perhaps outline the shape in black to make it stand out more?*

**Response**: We have tried outlining the structure in different colors but the results were the asterisk was removed and the structure was outlined in yellow to make it stand out.

*Page 7, paragraph after Fig. 3 caption - The auriculotemporal branch if V3 is mentioned in the second sentence of the paragraph, but it doesn’t appear to be labeled in Figure 3 unless I am missing it. Maybe move the citation for figure 4 up to this part of the paragraph?*

**Response**: Corrected. This should have been a reference to Figure 4, not 3. We have adjusted accordingly, with caption specified to indicate the auriculotemporal branch, number 1 (Fig 4, 1).

**Remaining Reviewer #1 comments (Fig 4 onward):**

*Figure 4 — The numbers in this figure appear to be off. For example, #9 is clearly pointing to the skull and not a nerve as identified in the caption. Also, #15 looks like the tympanic bulla rather than the external ear canal, which is either unlabeled, or perhaps #14? Lastly, I don’t see #16 in the figure, which the caption says should be the tympanic bone.*

**Response:** Thank you for catching this. All numeric labels in Figure 4 were replaced with lettered abbreviations and re-checked against the 3D reconstruction; the mismatched labels (facial nerve, the external ear canal / tympanic bulla, and the tympanic bone) were corrected and the caption updated accordingly.

*Figure 5 — Add number citations behind anatomical structures in the text of the caption to follow the style of other figure captions. Regarding the maxillary vein, the muscles are colored red also which makes the maxillary vein difficult to distinguish. Is #15 actually pointing to the tympanic bone? How is this distinguishable from the IMFB vascular plexus (#14)?*

**Response:** Number citations have been replaced with lettered abreviations in all figures. The maxillary vein, was actually mislabelled as it is considered the maxillary artery, as labelled in fig 1. This was also corrected in Figure 10 where it was previously mislabelled as well. The colour was changed to purple to make it better distinguishable. The IMFB and Tympanic bone indications have been corrected. The structure previously labelled ‘IMFB vascular plexus’ (Fig 5, #14) was renamed the intramandibular venous plexus (IVP), the standard term in the literature (Costidis & Rommel, 2012, and the two vascular papers in 2016), and this terminology is now applied consistently across the figures, captions and text (also replacing ‘mandibular fibrovenous plexus’ in Fig 10 and the descriptive label in Fig 3).

*Page 9, last sentence of 2nd paragraph after Fig. 5 caption — “…not visibly connect to the main plexus…” should be “…not visibly connected to the main plexus…”.*

**Response:** Adjusted accordingly (tracked change).

*Figure 6 — There don’t appear to be numbered structures in the image although they are referenced in the figure caption. Also, anti-DAP is stained blue, but there is a lot of blue in the image; is it all of the blue?*

**Response:** The numbered labels are made visible in the figure and more labels are added for epithelium and lamellar corpuscles. The caption now clarifies the anti-DAP1 (blue, nuclear) signal, which was also used in overexposed and desaturated mode to show the background structures.

*Figure 7 — I was confused by what I was looking at. What are the colours representing? Do the panels represent the same corpuscle at different focal depths? The caption states “five consecutive images”, although I see six (A–F); is there a reason A, C and E aren’t adjacent?*

**Response:** The caption is expanded to explain that the panels are consecutive sections through the same lamellar corpuscle, with two complementary stain combinations (A/C/E vs B/D/F), in which NF and DAPI are common, and only PGP9.5 and S100 are different because those two staining reactions cannot be applied in the same tissue cut. The meaning of each colour is clarified. The “five/six” discrepancy is corrected. As to why A/C/E are not adjacent, that is because the staining is alternating combinations: Cut A is stained with S100, the next cut (B) is stained with PGP9.5, C is S100, D is PGP9.5, E is S100, and F is PGP9.5. We opted for this alternate staining to get detailed views of the corpuscle throughout its course.

*Figure 8 — This is a cross section through the external ear canal, correct? What is the orientation (anterior, dorsal, etc.)? The red circles around the nervous structures are difficult to see (red on pink) and the individual ID numbers are illegible.*

**Response:** Yes, it is a transverse section through the external ear canal. The circles around the nervous structures were changed to black to improve their visibility against the pink background, and the individual ID numbers were removed, as they did not convey interpretable information. If the reviewers wish, we can provide a GIF/video showing the annotations throughout the complete stack of 50 sections as supporting information. Regarding orientation: at this level of the ear canal, this field of view contains no reference structures that would allow an unambiguous anatomical orientation (anterior, dorsal, etc.) to be assigned. At this level, the ear canal runs horizontal, so it is a lateral view of the cross-section, but it is not known which is side anterior/posterior or dorsal/ventral, and therefore not indicated.

*Figure 9 — This is an interesting image! Which direction is external?*

**Response:** Thank you! The direction now indicated in the figure and in the caption.

*Page 13, under ‘Accessory sinus system and vascular plexuses’ — are the trabeculated air spaces observable in any figures? If so, please include a figure citation. Also, the “1” in the section heading does not follow the style of other section headings.*

**Response:** The trabeculated air spaces are visible for a small part in Figure 1, in the peribullar sinus. However, that does not reflect the intricateness of the cranial sinuses which have not been described in detail in this manuscript as detailed descirptions can be found e.g. in Costidis and Rommel, 2012, where the authors focused on the relationship with vasculature.
The heading numbering has been removed to correspond with other section headings

*Figure 11 — Would it be possible to label some of the “large nerve fibres in the fibrovenous wall”?*

**Response:** The large nerve fibres in the fibrovenous wall have been labelled with asterisks, together with labels for the intramandibular fat body, pterygoid sinus, and the fibrovenous wall, in the figure and the caption.

*Page 15, first paragraph under ‘Discussion’ — it should be noted that the minke whale is a mysticete and that the anatomy observed in that species might be convergent with what is observed in odontocetes.*

**Response:** Agreed. The sentence now reads: *Yamato et al.* [*[33]*](https://www.zotero.org/google-docs/?gyyjG1) *documented analogous lateral fat bodies in the minke whale, a mysticete whale, providing anatomical evidence for convergent acoustic pathways that can vary considerably in geometry and functional role across lineages. However, caution should be applied before generalising lateral pathway models across cetaceans and, more specifically, odontocete taxa without species-specific acoustic and lipid biochemistry data.*

Reviewer #2: This is an interesting and useful paper with excellent figures supporting a detailed reconstruction of the peripheral auditory region of toothed whales. It corroborates prior research and adds meaningfully to our understanding of the region. I only have a few minor comments to improve clarity, particularly regarding the figures. Please see the attached annotated PDFs for specific notes.

**Response:** Thank you very much. Detailed replies are provided here below.

**Reviewer #2 — specific notes from the annotated PDFs:**

*Fig 1 — please indicate which taxon this is.*

**Response:** Done. The Fig 1 caption, and other captions, now specifies the striped dolphin (*Stenella coeruleoalba*).

*Fig 1 — label 7 seems to clearly be the periotic but not tympanic; it might be good to point out the tympanic bulla.*

**Response:** Labels for both bones added in the figure, and also clarified in the updated caption now: tympanic (TYB) and periotic (PER) bones.

*Fig 2 — the number ‘2’ is hard to see; can it be offset a bit more?*

**Response:** All numbers have been replaced with abbreviations throughout the entire manuscript for clarity.

*Fig 2 — ‘15. blue dot in Cranford (2010, Fig 10)’: please explain further so a reader can identify what it is.*

**Response:** Reference to Cranford (2010) has been removed and Structure 15 is explained in the caption as follows: Acoustic fat contacting in two places: the sigmoid process, and the malleus (MAL), directly contacting the ossicular chain.

*Fig 3 — for thoroughness, include the full genus and species name.*

**Response:** Done. The Fig 3 caption now reads ‘striped dolphin (*Stenella coeruleoalba*)’.

*Fig 3 — the yellow asterisk is difficult to see and pixelated; please remedy.*

**Response:** The asterisk was first replaced by a yellow outline but this made the image look strange and did not well define the location of the structure, so the yellow asterisk was maintained but with a larger size.

*Fig 3 — the images are a little blurry; the right-hand image looks like dissection material rather than a 3D CT reconstruction unless coloration was added.*

**Response:** The blurry images appear only in the reviewer pdf as it seems a low resolution document. The images are uploaded separately at full resolution. The right-hand side images is a 3D reconstruction with materials developed and added in Blender to achieve a more realistic look.

*Fig 4 — very realistic 3D reconstruction (from CT scans? photogrammetry? please specify), with even the coloration of the fat — or is it a dissection?*

**Response:** Thank you, indeed it is a 3D reconstruction based on DICE-µCT segmentation. The caption now read that the reconstruction derives from DICE-µCT segmentation.

*Fig 4 — ‘check this’ (facial nerve), ‘please check’ (mandible), and ‘all numbering from here is off, there may be a missing label’. A label doesn’t appear to point to the tympanic bone.*

**Response:** All labels have been updated with letter abbreviations for clarity, and the indicated mislabels were corrected.

*Fig 6 — if there are numbers in the figure, they are not visible.*

**Response:** The numbered labels are made visible.

*Caption (p19) — full species name would also be great.*

**Response:** Done. The full species name (*Stenella coeruleoalba*) has been added.

*Figs 9 and 10 — ‘This is an awesome image!’ / ‘Super cool.’*

**Response:** We thank the reviewer for the positive feedback.

*Text corrections marked in PONE-D-26-23707_RR.pdf.*

**Response:** The marked typographic corrections have been made as tracked changes: ‘out’→‘outer’, ‘connect’→‘connected’, ‘aroud’→‘around’, ‘in direction of’→‘in the direction of’, ‘further rostral’→‘positioned further rostral’, and ‘indiations’→‘indications’. The highlighted word ‘proven’ has been changed to ‘proven to exist’.
