# Supplementary material for: Peripheral anatomy of the dolphin ear and associated nervous structures: Insights from macroscopic dissection, DICE-µCT, histology, and confocal microscopy
Source: PLoS One. 2026 Aug 19;21(8):e0350484. doi: 10.1371/journal.pone.0350484 (PMC13489439; doi:10.1371/journal.pone.0350484)
Supplement: Peer review history

# Peer review: Decision Letter 0

**DOI:** 10.1371/journal.pone.0350484.r001

**Authors:** Michelangelo Bisconti

24 Jun 2026

PONE-D-26-23707

Peripheral anatomy of the dolphin ear and associated nervous structures: insights from macroscopic dissection, DICE-µCT, histology, and confocal microscopy

PLOS One

Dear Dr. De Vreese,

Thank you for submitting your manuscript to PLOS ONE. After careful consideration, we feel that it has merit but does not fully meet PLOS ONE’s publication criteria as it currently stands. Therefore, we invite you to submit a revised version of the manuscript that addresses the points raised during the review process.

==============================

**ACADEMIC EDITOR:**  Dear Dr. De Vreese,

I am writing to inform you that your manuscript titled Peripheral Anatomy of the Dolphin Ear and Associated Nervous Structures: Insights from Macroscopic Dissection, DICE-µCT, Histology, and Confocal Microscopy was reviewed and that both reviewers suggested minor revisions. The reviewers found it particularly difficult to interpret the figures, figure letterings and caption structures. Fundamentally, I share their opinion. Please, provide a revision of the manuscript by taking care of the reviewers’ suggestions and requests before resubmitting it.

==============================

Please submit your revised manuscript by Aug 09 2026 11:59PM. If you will need more time than this to complete your revisions, please reply to this message or contact the journal office at plosone@plos.org. When you're ready to submit your revision, log on to https://www.editorialmanager.com/pone/ and select the 'Submissions Needing Revision' folder to locate your manuscript file.

Please include the following items when submitting your revised manuscript:

- A letter that responds to each point raised by the academic editor and reviewer(s). You should upload this letter as a separate file labeled 'Response to Reviewers'.
- A marked-up copy of your manuscript that highlights changes made to the original version. You should upload this as a separate file labeled 'Revised Manuscript with Track Changes'.
- An unmarked version of your revised paper without tracked changes. You should upload this as a separate file labeled 'Manuscript'.

If you would like to make changes to your financial disclosure, please include your updated statement in your cover letter. Guidelines for resubmitting your figure files are available below the reviewer comments at the end of this letter.

If applicable, we recommend that you deposit your laboratory protocols in protocols.io to enhance the reproducibility of your results. Protocols.io assigns your protocol its own identifier (DOI) so that it can be cited independently in the future. For instructions see: https://journals.plos.org/plosone/s/submission-guidelines#loc-laboratory-protocols. Additionally, PLOS ONE offers an option for publishing peer-reviewed Lab Protocol articles, which describe protocols hosted on protocols.io. Read more information on sharing protocols at https://plos.org/protocols?utm_medium=editorial-email&utm_source=authorletters&utm_campaign=protocols.

As the corresponding author, your ORCID iD is verified in the submission system and will appear in the published article. PLOS supports the use of ORCID, and we encourage all coauthors to register for an ORCID iD and use it as well. Please encourage your coauthors to verify their ORCID iD within the submission system before final acceptance, as unverified ORCID iDs will not appear in the published article. *Only*  the individual author can complete the verification step; PLOS staff *cannot*  verify ORCID iDs on behalf of authors.

We look forward to receiving your revised manuscript.

Kind regards,

Michelangelo Bisconti, PhD

Academic Editor

PLOS One

Journal requirements:

When submitting your revision, we need you to address these additional requirements.

1. Please ensure that your manuscript meets PLOS ONE's style requirements, including those for file naming. The PLOS ONE style templates can be found at

https://journals.plos.org/plosone/s/file?id=wjVg/PLOSOne_formatting_sample_main_body.pdf and

https://journals.plos.org/plosone/s/file?id=ba62/PLOSOne_formatting_sample_title_authors_affiliations.pdf

2. Please note that PLOS One has specific guidelines on code sharing for submissions in which author-generated code underpins the findings in the manuscript. In these cases, we expect all author-generated code to be made available without restrictions upon publication of the work. Please review our guidelines at https://journals.plos.org/plosone/s/materials-and-software-sharing#loc-sharing-code and ensure that your code is shared in a way that follows best practice and facilitates reproducibility and reuse.

3. Thank you for stating the following financial disclosure:

“Part of this study was funded by the Italian Cassa di Risparmio del Veneto, while another as part of the Project PCI2022-135022-2 financed by MICIU/AEI /10.13039/501100011033 and by the European Union NextGenerationEU/PRTR, supported by JPI Oceans.”

Please state what role the funders took in the study.  If the funders had no role, please state: "The funders had no role in study design, data collection and analysis, decision to publish, or preparation of the manuscript."

If this statement is not correct you must amend it as needed.

Please include this amended Role of Funder statement in your cover letter; we will change the online submission form on your behalf.

4. Thank you for stating the following in the Acknowledgments Section of your manuscript:

“Part of this study was funded by the Italian Fondazione Cassa di Risparmio del Veneto e Rovigo, while another as part of the Project PCI2022-135022-2 financed by MICIU/AEI /10.13039/501100011033 and by the European Union NextGenerationEU/PRTR, supported by JPI Oceans. Gmar is funded by a Catalan Government Grups de Recerca Consolidats grant (ref. 2021 SGR 01195). The authors would like to acknowledge the Italian Zooprofilactic Institutes of Venezia, Lazio e Toscana, Lìguria, and Sardinia for collaboration in the sampling network, and Mariano Domingo (Department of Health and Anatomy, Faculty of Veterinary Medicine, Autonomous University of Barcelona, Bellaterra). Also thanks to Prof. dr. Hansjörg Schröder of the Department II of Anatomy (Neuroanatomy), University of Cologne (50924 Cologne, Germany) for allowing SDV to perform part of the macroscopic studies on site.”

We note that you have provided funding information that is currently declared in your Funding Statement. However, funding information should not appear in the Acknowledgments section or other areas of your manuscript. We will only publish funding information present in the Funding Statement section of the online submission form.

Please remove any funding-related text from the manuscript and let us know how you would like to update your Funding Statement. Currently, your Funding Statement reads as follows:

“Part of this study was funded by the Italian Cassa di Risparmio del Veneto, while another as part of the Project PCI2022-135022-2 financed by MICIU/AEI /10.13039/501100011033 and by the European Union NextGenerationEU/PRTR, supported by JPI Oceans.”

Please include your amended statements within your cover letter; we will change the online submission form on your behalf.

5. In the online submission form you indicate that your data is not available for proprietary reasons and have provided a contact point for accessing this data. Please note that your current contact point is a co-author on this manuscript. According to our Data Policy, the contact point must not be an author on the manuscript and must be an institutional contact, ideally not an individual. Please revise your data statement to a non-author institutional point of contact, such as a data access or ethics committee, and send this to us via return email. Please also include contact information for the third party organization, and please include the full citation of where the data can be found.

6. We notice that your supplementary figures are uploaded with the file type 'Figure'. Please amend the file type to 'Supporting Information'. Please ensure that each Supporting Information file has a legend listed in the manuscript after the references list.

If the reviewer comments include a recommendation to cite specific previously published works, please review and evaluate these publications to determine whether they are relevant and should be cited. There is no requirement to cite these works unless the editor has indicated otherwise.

Please review your reference list to ensure that it is complete and correct. If you have cited papers that have been retracted, please include the rationale for doing so in the manuscript text, or remove these references and replace them with relevant current references. Any changes to the reference list should be mentioned in the rebuttal letter that accompanies your revised manuscript. If you need to cite a retracted article, indicate the article’s retracted status in the References list and also include a citation and full reference for the retraction notice.

[Note: HTML markup is below. Please do not edit.]

Reviewers' comments:

Reviewer's Responses to Questions

**Comments to the Author**

1. Is the manuscript technically sound, and do the data support the conclusions?

The manuscript must describe a technically sound piece of scientific research with data that supports the conclusions. Experiments must have been conducted rigorously, with appropriate controls, replication, and sample sizes. The conclusions must be drawn appropriately based on the data presented.

Reviewer #1: Yes

Reviewer #2: Yes

**********

2. Has the statistical analysis been performed appropriately and rigorously?

Reviewer #1: Yes

Reviewer #2: N/A

**********

3. Have the authors made all data underlying the findings in their manuscript fully available?

The PLOS Data policy requires authors to make all data underlying the findings described in their manuscript fully available without restriction, with rare exception (please refer to the Data Availability Statement in the manuscript PDF file). The data should be provided as part of the manuscript or its supporting information, or deposited to a public repository. For example, in addition to summary statistics, the data points behind means, medians and variance measures should be available. If there are restrictions on publicly sharing data—e.g. participant privacy or use of data from a third party—those must be specified.

Reviewer #1: Yes

Reviewer #2: Yes

**********

4. Is the manuscript presented in an intelligible fashion and written in standard English?

PLOS ONE does not copyedit accepted manuscripts, so the language in submitted articles must be clear, correct, and unambiguous. Any typographical or grammatical errors should be corrected at revision, so please note any specific errors here.

Reviewer #1: Yes

Reviewer #2: Yes

**********

5. Review Comments to the Author

Please use the space provided to explain your answers to the questions above. You may also include additional comments for the author, including concerns about dual publication, research ethics, or publication ethics. (Please upload your review as an attachment if it exceeds 20,000 characters)

Reviewer #1: I found the paper to be very interesting and a much needed description of the outer ear of odontocetes, which is an anatomical region that has received little attention. I particularly appreciate the discussion of the potential function of the outer ear region, which often is considered functionless.

Overall, the paper is well written and the conclusions are well supported by the observations presented. The figures are good, although I did find some of them a little difficult to interpret. In fact, the vast majority of my comments deal with the figures and associated captions themselves. I have made several comments and suggestions below, both general and specific, all of which I think will strengthen the paper.

A general comment about the figures is that a lot of the anatomy is difficult to interpret. Figures 4 & 5 were probably the most helpful, but I think that an illustrated or schematic figure that represents the 3-dimensional shapes would be very helpful. I also found the numbers in the figure to be a bit distracting and it was a little cumbersome to flick back and forth between the figure and the caption. One option would be to replace them with abbreviations, but that might be a matter of taste. I found the numbering to be especially distracting when they aren’t all listed numerically at the end of the captions, such as in Figure 3 in which some of the structures are numbered in the text of the caption. I would recommend listing all of the numbers at the end of the caption even if they are listed earlier in the caption text.

I also think that more specific citations to the figures would help the reader to follow the anatomical descriptions, especially in the ‘Mandibular fat bodies’ section beginning around page 11. When possible, please cite the figures more frequently.

Page 3, second sentence under ‘Materials and Methods’ - I think that “spotted dolphin foetus" should be plural here. (E.g., “two bottlenose dolphin…and Pantropical spotted dolphin foetuses.”)

Further in the same paragraph, “details” in “details can be found” should be lowercase.

First sentence of third paragraph under ‘Material and Methods’, “from the out ear canal” should be “from the outer ear canal”.

Page 5, first paragraph under ‘Region of the ear canal and middle ear’ - the anatomy described here is difficult to interpret even using the figures. This place in particular would benefit from a schematic or illustrated figure to orient the reader and accentuate the anatomy.

Page 6, Figure 1 caption - In case I missed it, IMFB and EMFB have not been defined by this point in the manuscript. These should be defined (i.e., spelled out) earlier in the manuscript.

Page 6, Figure 2 caption - Define/ spell out “TP-complex”. I am guessing this is the tympanoperiotic complex.

Figure 3 - It could be on account of the resolution for the review, but the yellow asterisk was difficult to see. Perhaps outline the shape in black to make it stand out more?

Page 7, paragraph after Fig. 3 caption - The auriculotemporal branch if V3 is mentioned in the second sentence of the paragraph, but it doesn’t appear to be labeled in Figure 3 unless I am missing it. Maybe move the citation for figure 4 up to this part of the paragraph?

Figure 4 - The numbers in this figure appear to be off. For example, #9 is clearly pointing to the skull and not a nerve as identified in the caption. Also, #15 looks like the tympanic bulla rather than the external ear canal, which is either unlabeled, or perhaps #14? Lastly, I don’t see #16 in the figure, which the caption says should be the tympanic bone.

Figure 5 - Add number citations behind anatomical structures in the text of the caption to follow the style of other figure captions. Regarding the maxillary vein, the muscles are colored red also which makes the maxillary vein difficult to distinguish. Is #15 actually pointing to the tympanic bone? How is this distinguishable from the IMFB vascular plexus (#14)?

Page 9, last sentence of 2nd paragraph after Fig. 5 caption - “…not visibly connect to the main plexus…” should be “…not visibly connected to the main plexus…”

Figure 6 - There don’t appear to be numbered structures in the image although they are referenced in the figure caption. Also, anti-DAP2 is stained blue, but there is a lot of blue in the image. Is it all of the blue? I have to admit that I am not very well versed in confocal microscopy or staining techniques involved, and so I had difficulty following much of this.

Figure 7 - Following my comment above, I was confused by what I was looking at in this figure and so further explanation of what is being presented would be helpful. What are the colors representing? Do each of the panels represent the same corpuscle just at different focal depths? The caption states that there are “five consecutive images”, although I see a total of six (A, B, C, D, E, & F). It appears that we are to compare panels A, C, and E. If so, is there a reason that they aren’t adjacent to one another? This may relate to my ignorance of confocal microscopy techniques, but I was generally confused by this figure.

Figure 8 - This is a cross section through the external ear canal, correct? What is the orientation (anterior, dorsal, etc.)? The red circles around the nervous structures are difficult to see (red on pink) and the individual ID numbers are illegible.

Figure 9 - This is an interesting image! Which direction is external?

Page 13, under ‘1. Accessory sinus system and vascular plexuses’ - are the trabeculated air spaces observable in any figures? If so, please include a figure citation. Also, the “1” in the section heading does not follow the style of other section headings.

Figure 11 - Would it be possible to label some of the “large nerve fibers in the fibrovenous wall?”

Page 15, first paragraph under ‘Discussion’ - It should be noted that the minke whale is a mysticete and that the anatomy observed in that species might be convergent with what is observed in odontocetes.

Reviewer #2: This is an interesting and useful paper with excellent figures supporting a detailed reconstruction of the peripheral auditory region of toothed whales. It corroborates prior research and adds meaningfully to our understanding of the region. I only have a few minor comments to improve clarity, particularly regarding the figures. Please see the attached annotated PDFs for specific notes.

**********

6. PLOS authors have the option to publish the peer review history of their article (what does this mean?). If published, this will include your full peer review and any attached files.

If you choose “no”, your identity will remain anonymous but your review may still be made public.

**Do you want your identity to be public for this peer review?**  For information about this choice, including consent withdrawal, please see our Privacy Policy.

Reviewer #1: **Yes:** Eric G. Ekdale

Reviewer #2: No

**********

[NOTE: If reviewer comments were submitted as an attachment file, they will be attached to this email and accessible via the submission site. Please log into your account, locate the manuscript record, and check for the action link "View Attachments". If this link does not appear, there are no attachment files.]

To ensure your figures meet our technical requirements, please review our figure guidelines: https://journals.plos.org/plosone/s/figures

You may also use PLOS’s free figure tool, NAAS, to help you prepare publication quality figures: https://journals.plos.org/plosone/s/figures#loc-tools-for-figure-preparation.

NAAS will assess whether your figures meet our technical requirements by comparing each figure against our figure specifications.

Attachment Submitted filename: PONE-D-26-23707_RR.pdf

Attachment Submitted filename: PONE-D-26-23707_reviewer-Fig_captions.pdf

# Author comment: Author response to Decision Letter 1

**DOI:** 10.1371/journal.pone.0350484.r002

6 Jul 2026

Answer to editor comments:

Financial Disclosure

Part of this study was funded by the Italian Fondazione Cassa di Risparmio del Veneto e Rovigo (awarded to SDV), while another as part of the Project PCI2022-135022-2 financed by MCIN/AEI /10.13039/501100011033 and by the European Union NextGenerationEU/PRTR, supported by JPI Oceans (awarded to SDV; https://www.aei.gob.es/ ; https://www.jpi-oceans.eu/). Gmar is funded by a Catalan Government Grups de Recerca Consolidats grant (ref. 2021 SGR 01195) (awarded to MCD and JF; https://agaur.gencat.cat/). The funders had no role in study design, data collection and analysis, decision to publish, or preparation of the manuscript.

Role of the Funders

The funders had no role in study design, data collection and analysis, decision to publish, or preparation of the manuscript.

We confirm that all funding-related information has been removed from the Acknowledgments section and now appears only in the Funding Statement provided here.

Regarding point 5 in your email: Data Availability

All data underlying the findings are within the manuscript and its Supporting Information files. The raw DICE-µCT imaging and derived 3D reconstruction files, histology files, and other tissues are of protected cetacean species and were obtained under specific use agreements with the providing institutions; they therefore cannot be shared publicly owing to third-party ownership and permit restrictions.

Regarding point 6 in your email: Figures vs Supporting Information

My apologies but it is not clear what is intended here. All figures are part of the manuscript and not supplementary information. However, I followed the instructions and amend the file type to Supporting Information. All have a legend at the end of the manuscript after the references list.

Answer to reviewers

Reviewer #1: I found the paper to be very interesting and a much needed description of the outer ear of odontocetes, which is an anatomical region that has received little attention. I particularly appreciate the discussion of the potential function of the outer ear region, which often is considered functionless.

Overall, the paper is well written and the conclusions are well supported by the observations presented. The figures are good, although I did find some of them a little difficult to interpret. In fact, the vast majority of my comments deal with the figures and associated captions themselves. I have made several comments and suggestions below, both general and specific, all of which I think will strengthen the paper.

A general comment about the figures is that a lot of the anatomy is difficult to interpret. Figures 4 & 5 were probably the most helpful, but I think that an illustrated or schematic figure that represents the 3-dimensional shapes would be very helpful. I also found the numbers in the figure to be a bit distracting and it was a little cumbersome to flick back and forth between the figure and the caption. One option would be to replace them with abbreviations, but that might be a matter of taste. I found the numbering to be especially distracting when they aren’t all listed numerically at the end of the captions, such as in Figure 3 in which some of the structures are numbered in the text of the caption. I would recommend listing all of the numbers at the end of the caption even if they are listed earlier in the caption text.

I also think that more specific citations to the figures would help the reader to follow the anatomical descriptions, especially in the ‘Mandibular fat bodies’ section beginning around page 11. When possible, please cite the figures more frequently.

Response: Thank you for the suggestions, indeed it is a complex anatomical region that requires simplification of 3-dimensional shapes to guide the reader for orientation and identification of the anatomical structures of interest. We have added a simplified drawing of the anatomical components from the DICE-µCT study. We hope this helps for clarification of the topographical relationship between fat bodies and nerves. Also, the entire manuscript has been updated to include a single naming convention for each structure, to avoid the reader to hop back and forth for number identification in the figures. Additional citations to figures have been added throughout the text, and in the mandibular fat body section.

Page 3, second sentence under ‘Materials and Methods’ - I think that “spotted dolphin foetus" should be plural here. (E.g., “two bottlenose dolphin…and Pantropical spotted dolphin foetuses.”)

Response: That macroscopic dissection sample came from a single spotted dolphin foetus; sentence has been adjusted accordingly; and ‘single’ was removed from the last sentence of the paragraph to avoid repetition.

Further in the same paragraph, “details” in “details can be found” should be lowercase.

Response: Adjusted accordingly.

First sentence of third paragraph under ‘Material and Methods’, “from the out ear canal” should be “from the outer ear canal”.

Response: Adjusted accordingly.

Page 5, first paragraph under ‘Region of the ear canal and middle ear’ - the anatomy described here is difficult to interpret even using the figures. This place in particular would benefit from a schematic or illustrated figure to orient the reader and accentuate the anatomy.

Response: An illustrated simplified figure has been added to orient the reader, Figure 05.

Page 6, Figure 1 caption - In case I missed it, IMFB and EMFB have not been defined by this point in the manuscript. These should be defined (i.e., spelled out) earlier in the manuscript.

Response: Thank you for pointing this out. The spelled-out words and abbreviations have been added to the paragraph before Figure 1, with specific references to the structure identifications in the figure.

Page 6, Figure 2 caption - Define/ spell out “TP-complex”. I am guessing this is the tympanoperiotic complex.

Response: TP-complex is now spelled out, with abbreviation ‘TPC’, and also adjusted throughout the manuscript.

Figure 3 - It could be on account of the resolution for the review, but the yellow asterisk was difficult to see. Perhaps outline the shape in black to make it stand out more?

Response: We have tried outlining the structure in different colors but the results were the asterisk was removed and the structure was outlined in yellow to make it stand out.

Page 7, paragraph after Fig. 3 caption - The auriculotemporal branch if V3 is mentioned in the second sentence of the paragraph, but it doesn’t appear to be labeled in Figure 3 unless I am missing it. Maybe move the citation for figure 4 up to this part of the paragraph?

Response: Corrected. This should have been a reference to Figure 4, not 3. We have adjusted accordingly, with caption specified to indicate the auriculotemporal branch, number 1 (Fig 4, 1).

Remaining Reviewer #1 comments (Fig 4 onward):

Figure 4 — The numbers in this figure appear to be off. For example, #9 is clearly pointing to the skull and not a nerve as identified in the caption. Also, #15 looks like the tympanic bulla rather than the external ear canal, which is either unlabeled, or perhaps #14? Lastly, I don’t see #16 in the figure, which the caption says should be the tympanic bone.

Response: Thank you for catching this. All numeric labels in Figure 4 were replaced with lettered abbreviations and re-checked against the 3D reconstruction; the mismatched labels (facial nerve, the external ear canal / tympanic bulla, and the tympanic bone) were corrected and the caption updated accordingly.

Figure 5 — Add number citations behind anatomical structures in the text of the caption to follow the style of other figure captions. Regarding the maxillary vein, the muscles are colored red also which makes the maxillary vein difficult to distinguish. Is #15 actually pointing to the tympanic bone? How is this distinguishable from the IMFB vascular plexus (#14)?

Response: Number citations have been replaced with lettered abreviations in all figures. The maxillary vein, was actually mislabelled as it is considered the maxillary artery, as labelled in fig 1. This was also corrected in Figure 10 where it was previously mislabelled as well. The colour was changed to purple to make it better distinguishable. The IMFB and Tympanic bone indications have been corrected. The structure previously labelled ‘IMFB vascular plexus’ (Fig 5, #14) was renamed the intramandibular venous plexus (IVP), the standard term in the literature (Costidis & Rommel, 2012, and the two vascular papers in 2016), and this terminology is now applied consistently across the figures, captions and text (also replacing ‘mandibular fibrovenous plexus’ in Fig 10 and the descriptive label in Fig 3).

Page 9, last sentence of 2nd paragraph after Fig. 5 caption — “…not visibly connect to the main plexus…” should be “…not visibly connected to the main plexus…”.

Response: Adjusted accordingly (tracked change).

Figure 6 — There don’t appear to be numbered structures in the image although they are referenced in the figure caption. Also, anti-DAP is stained blue, but there is a lot of blue in the image; is it all of the blue?

Response: The numbered labels are made visible in the figure and more labels are added for epithelium and lamellar corpuscles. The caption now clarifies the anti-DAP1 (blue, nuclear) signal, which was also used in overexposed and desaturated mode to show the background structures.

Figure 7 — I was confused by what I was looking at. What are the colours representing? Do the panels represent the same corpuscle at different focal depths? The caption states “five consecutive images”, although I see six (A–F); is there a reason A, C and E aren’t adjacent?

Response: The caption is expanded to explain that the panels are consecutive sections through the same lamellar corpuscle, with two complementary stain combinations (A/C/E vs B/D/F), in which NF and DAPI are common, and only PGP9.5 and S100 are different because those two staining reactions cannot be applied in the same tissue cut. The meaning of each colour is clarified. The “five/six” discrepancy is corrected. As to why A/C/E are not adjacent, that is because the staining is alternating combinations: Cut A is stained with S100, the next cut (B) is stained with PGP9.5, C is S100, D is PGP9.5, E is S100, and F is PGP9.5. We opted for this alternate staining to get detailed views of the corpuscle throughout its course.

Figure 8 — This is a cross section through the external ear canal, correct? What is the orientation (anterior, dorsal, etc.)? The red circles around the nervous structures are difficult to see (red on pink) and the individual ID numbers are illegible.

Response: Yes, it is a transverse section through the external ear canal. The circles around the nervous structures were changed to black to improve their visibility against the pink background, and the individual ID numbers were removed, as they did not convey interpretable information. If the reviewers wish, we can provide a GIF/video showing the annotations throughout the complete stack of 50 sections as supporting information. Regarding orientation: at this level of the ear canal, this field of view contains no reference structures that would allow an unambiguous anatomical orientation (anterior, dorsal, etc.) to be assigned. At this level, the ear canal runs horizontal, so it is a lateral view of the cross-section, but it is not known which is side anterior/posterior or dorsal/ventral, and therefore not indicated.

Figure 9 — This is an interesting image! Which direction is external?

Response: Thank you! The direction now indicated in the figure and in the caption.

Page 13, under ‘Accessory sinus system and vascular plexuses’ — are the trabeculated air spaces observable in any figures? If so, please include a figure citation. Also, the “1” in the section heading does not follow the style of other section headings.

Response: The trabeculated air spaces are visible for a small part in Figure 1, in the peribullar sinus. However, that does not reflect the intricateness of the cranial sinuses which have not been described in detail in this manuscript as detailed descirptions can be found e.g. in Costidis and Rommel, 2012, where the authors focused on the relationship with vasculature.

The heading numbering has been removed to correspond with other section headings

Figure 11 — Would it be possible to label some of the “large nerve fibres in the fibrovenous wall”?

Response: The large nerve fibres in the fibrovenous wall have been labelled with asterisks, together with labels for the intramandibular fat body, pterygoid sinus, and the fibrovenous wall, in the figure and the caption.

Page 15, first paragraph under ‘Discussion’ — it should be noted that the minke whale is a mysticete and that the anatomy observed in that species might be convergent with what is observed in odontocetes.

Response: Agreed. The sentence now reads: Yamato et al. [33] documented analogous lateral fat bodies in the minke whale, a mysticete whale, providing anatomical evidence for convergent acoustic pathways that can vary considerably in geometry and functional role across lineages. However, caution should be applied before generalising lateral pathway models across cetaceans and, more specifically, odontocete taxa without species-specific acoustic and lipid biochemistry data.

Reviewer #2: This is an interesting and useful paper with excellent figures supporting a detailed reconstruction of the peripheral auditory region of toothed whales. It corroborates prior research and adds meaningfully to our understanding of the region. I only have a few minor comments to improve clarity, particularly regarding the figures. Please see the attached annotated PDFs for specific notes.

Response: Thank you very much. Detailed replies are provided here below.

Reviewer #2 — specific notes from the annotated PDFs:

Fig 1 — please indicate which taxon this is.

Response: Done. The Fig 1 caption, and other captions, now specifies the striped dolphin (Stenella coeruleoalba).

Fig 1 — label 7 seems to clearly be the periotic but not tympanic; it might be good to point out the tympanic bulla.

Response: Labels for both bones added in the figure, and also clarified in the updated caption now: tympanic (TYB) and periotic (PER) bones.

Fig 2 — the number ‘2’ is hard to see; can it be offset a bit more?

Response: All numbers have been replaced with abbreviations throughout the entire manuscript for clarity.

Fig 2 — ‘15. blue dot in Cranford (2010, Fig 10)’: please explain further so a reader can identify what it is.

Response: Reference to Cranford (2010) has been removed and Structure 15 is explained in the caption as follows: Acoustic fat contacting in two places: the sigmoid process, and the malleus (MAL), directly contacting the ossicular chain.

Fig 3 — for thoroughness, include the full genus and species name.

Response: Done. The Fig 3 caption now reads ‘striped dolphin (Stenella coeruleoalba)’.

Fig 3 — the yellow asterisk is difficult to see and pixelated; please remedy.

Response: The asterisk was first replaced by a yellow outline but this made the image look strange and did not well define the location of the structure, so the yellow asterisk was maintained but with a larger size.

Fig 3 — the images are a little blurry; the right-hand image looks like dissection material rather than a 3D CT reconstruction unless coloration was added.

Response: The blurry images appear only in the reviewer pdf as it seems a low resolution document. The images are uploaded separately at full resolution. The right-hand side images is a 3D reconstruction with materials developed and added in Blender to achieve a more realistic look.

Fig 4 — very realistic 3D reconstruction (from CT scans? photogrammetry? please specify), with even the coloration of the fat — or is it a dissection?

Response: Thank you, indeed it is a 3D reconstruction based on DICE-µCT segmentation. The caption now read that the reconstruction derives from DICE-µCT segmentation.

Fig 4 — ‘check this’ (facial nerve), ‘please check’ (mandible), and ‘all numbering from here is off, there may be a missing label’. A label doesn’t appear to point to the tympanic bone.

Response: All labels have

Attachment Submitted filename: AnswerToReviewers_final.docx

# Editor report: Decision Letter 1

**DOI:** 10.1371/journal.pone.0350484.r003

**Authors:** Michelangelo Bisconti

14 Jul 2026

Peripheral anatomy of the dolphin ear and associated nervous structures: insights from macroscopic dissection, DICE-µCT, histology, and confocal microscopy

PONE-D-26-23707R1

Dear Dr. De Vreese,

We’re pleased to inform you that your manuscript has been judged scientifically suitable for publication and will be formally accepted for publication once it meets all outstanding technical requirements.

Within one week, you’ll receive an e-mail detailing the required amendments. When these have been addressed, you’ll receive a formal acceptance letter and your manuscript will be scheduled for publication.

An invoice will be generated when your article is formally accepted. Please note, if your institution has a publishing partnership with PLOS and your article meets the relevant criteria, all or part of your publication costs will be covered. Please make sure your user information is up-to-date by logging into Editorial Manager at Editorial Manager® and clicking the ‘Update My Information' link at the top of the page. For questions related to billing, please contact billing support.

If your institution or institutions have a press office, please notify them about your upcoming paper to help maximize its impact. If they’ll be preparing press materials, please inform our press team as soon as possible -- no later than 48 hours after receiving the formal acceptance. Your manuscript will remain under strict press embargo until 2 pm Eastern Time on the date of publication. For more information, please contact onepress@plos.org.

Kind regards,

Michelangelo Bisconti, PhD

Academic Editor

PLOS One

Additional Editor Comments (optional):

Dear Dr De Vreese,

I carefully read the revised version of your manuscript and the new illustrations together with the responses you and your co-authors provided to reviewers' questions and comments. I found that your revision is adequate and that the manuscript is now improved by addressing all the reviewers' questions. I therefore recommend that this manuscript can be accepted for publication in Plos ONE.

Congratulations and best regards,

Michelangelo Bisconti

Reviewers' comments:

# Editor report: Acceptance letter

**DOI:** 10.1371/journal.pone.0350484.r004

**Authors:** Michelangelo Bisconti

PONE-D-26-23707R1

PLOS One

Dear Dr. De Vreese,

I'm pleased to inform you that your manuscript has been deemed suitable for publication in PLOS One. Congratulations! Your manuscript is now being handed over to our production team.

At this stage, our production department will prepare your paper for publication. This includes ensuring the following:

* All references, tables, and figures are properly cited

* All relevant supporting information is included in the manuscript submission,

* There are no issues that prevent the paper from being properly typeset

You will receive further instructions from the production team, including instructions on how to review your proof when it is ready. Please keep in mind that we are working through a large volume of accepted articles, so please give us a few days to review your paper and let you know the next and final steps.

Lastly, if your institution or institutions have a press office, please let them know about your upcoming paper now to help maximize its impact. If they'll be preparing press materials, please inform our press team within the next 48 hours. Your manuscript will remain under strict press embargo until 2 pm Eastern Time on the date of publication. For more information, please contact onepress@plos.org.

You will receive an invoice from PLOS for your publication fee after your manuscript has reached the completed accept phase. If you receive an email requesting payment before acceptance or for any other service, this may be a phishing scheme. Learn how to identify phishing emails and protect your accounts at https://explore.plos.org/phishing.

If we can help with anything else, please email us at customercare@plos.org.

Thank you for submitting your work to PLOS ONE and supporting open access.

Kind regards,

PLOS ONE Editorial Office Staff

on behalf of

Doctor Michelangelo Bisconti

Academic Editor

PLOS One
